# Physicians' messages in problematic sickness certification: a narrative analysis of case reports

**DOI:** 10.1186/1471-2296-12-18

**Published:** 2011-04-11

**Authors:** Monika Engblom, Kristina Alexanderson, Carl Edvard Rudebeck

**Affiliations:** 1 Division of Insurance Medicine, Department of Clinical Neuroscience, Karolinska Institutet, Stockholm, Sweden; 2 Institute of Community Medicine, University of Tromsœ, Norway, and Research Unit, Kalmar County Council, Sweden

## Abstract

**Background:**

Many physicians find sickness certification tasks problematic. There is some knowledge about situations that are experienced as problematic, whereas less is understood about how physicians respond to the problems they face. One way to acquire such knowledge is to consider "reflection-in-action", aspects of which are expressed in the physician's interpretation of the patient's story. The aim of this study was to gain knowledge about the meaning content of case reports about problematic sickness certification. Specifically, we looked for possible messages to the colleagues intended to read the reports.

**Methods:**

A narrative approach was used to analyse reports about problematic sickness certification cases that had been written by GPs and occupational health service physicians as part of a sickness insurance course. The analysis included elements from both thematic and structural analysis. Nineteen case reports were used in the actual analysis and 25 in the validation of the results. Main narrative qualities and structural features of the written case reports were explored.

**Results:**

Five types of messages were identified in the case reports, here classified as "a call for help", "a call for understanding", "hidden worries", "in my opinion", and "appearing neutral". In the reports, the physicians tried to achieve neutrality in their writing, and the patients' stories tended to be interpreted within a traditional biomedical framework. In some cases there was an open request for help, in others it was not obvious that the physician had any problems. Overall, the messages were about having problems as such, rather than the specific features of the problems.

**Conclusions:**

The case reports clearly demonstrated different ways of writing about problems that arise during sickness certification, from being neutral and not mentioning the problems to being emotionally involved and asking for help. The general character of the messages suggests that they are also relevant for case reports in problematic areas other than sickness certification. If pertinent relationships can be found between reflection-in-practice and the narrative writing about practice, they will provide an approach to further research concerning consultations perceived as problematic and also to medical education.

## Background

Among the consultations that physicians perceive as problematic [1], a large proportion concern musculoskeletal or mental diagnoses [2,3], and such diagnoses also predominate on the sickness certificates issued in Western countries [4-8]. Indeed, many physicians find sickness certification problematic [7-10], especially when the decisions that are made must be based solely on the patients' descriptions of their symptoms [11,12], and the physicians also tend to be influenced by how the patients give these descriptions [13-15].

In previous qualitative studies [11,16], we identified different categories of dilemmas experienced by physicians in their handling of sickness certification. These difficulties arose under circumstances such as the following: when a patient's problem was judged to be non-medical in character; when there was a discrepancy between the patient's presentation of his/her symptoms and the physician's comprehension of them; when the physician perceived sickness certification per se as harmful. Furthermore, within a narrative framework, we previously conducted descriptive analyses of physicians' written case reports about sickness certification cases that they perceived as problematic [17]. In that study, we found that physicians described the following common characteristics for their patients: family situation, occupation, stressful life events, medical investigations, duration of sick leave, and treatments received. Prolonged sick leave appeared to be more or less inevitable in these cases. Other circumstances, such as patients' stressful life events, more closely reflected what the reporting physicians found to be problematic. From a narrative perspective, we learned that it takes a physician to make a case problematic.

To some extent, research has identified aspects of sickness certification that physicians consider to be problematic, and it is also recognized that these can differ between physicians [11,18], whereas very little is known about how physicians respond to the problems that they experience. One way to acquire such knowledge is to consider how these professionals think in practice. There are different methods for obtaining such information. In the book entitled "The Reflective Practitioner: How Professionals Think in Action", Schön [19] delineates the crucial role of reflection-in-action for the competence and professional behaviour of the practitioner. Reflection-in-action includes framing a problem in ways that provide options for action, and then, while still in action, determining whether the framing also provides solutions. The book "Doctors' Stories: The Narrative Structure of Medical Knowledge" written by Hunter [20] describes investigation of how physicians make sense of their medical cases. Hunter regards professional interaction with patients as an art that relies on interpreting the patient's story, and it is this interpretation that is the medical practitioner's mode of framing. One version of the interpretation is the conventional medical chart, and another is the case report, but, as Hunter put it, both these are "narrowly conceived and standardized by strict conventions of tone, plot, and allowable detail". Nonetheless, inevitably integrated in a case report is its explicit or implicit message to the reader, who is most often a colleague. In that perspective, framing of the message may coincide with creating one's identity as a physician and also with conveying knowledge or asking for advice [21].

The aim of the present study was to explore the meaning content of case reports about problematic sickness certification. Specifically, we looked for possible messages to the colleagues intended to read the reports.

The Regional Ethics Committee of Stockholm approved the study (Reg. no. 2005/760-31/4).

## Methods

Case reports written by physicians about problems experienced in connection with sickness certification were subjected to narrative analysis. This included thematic analysis as well as structural analysis.

### The physicians and the case reports

The case reports used here were written as a pre-assignment for participation in a five-day course designed to help physicians improve their skills in sickness certification [16]. Case reports from ten consecutive courses were used. Two hundred sixty physicians participated in the courses, which were held in different parts of Sweden from September 2006 to February 2008. About half of the participants were employed in occupational health services and nearly half as general practitioners (GPs), and a few worked in a rehabilitation clinic; 45% were women. Most of the occupational health service physicians were also board-certified specialists in general practice, psychiatry, orthopaedics, or internal medicine.

The physicians were asked to choose a case in which they had felt uncomfortable with their role in sickness certification and to write approximately one page about that particular case. They were also instructed to try to describe how they perceived such problems at the beginning of their careers and also at present (i.e., at the time a course was held). These written case reports were subsequently used in group discussions and role-play sessions in the courses.

Before the start of each sickness insurance course, written information about our study was provided, which requested the participants to specify whether or not their case report could be included in our analysis; all participants gave their consent. The case reports were numbered to correspond with the list of course participants.

The case reports varied in length from a few lines to two whole pages. To obtain a mixed selection of the material, we created a sample comprising a total of 20 case reports by using case numbers five and fifteen from each course. Unfortunately, one case report had to be excluded because it was incomplete, which left 19 reports for the analysis. The 19 physicians who wrote the reports were as follows: 11 worked in primary health care, five in occupational health care, and three in a rehabilitation clinic; six were women and 13 were men, with an average age of 52.3 years (30-63 years). An additional 25 case reports were analysed for validation of the results.

### The analysis

Narrative research concerns both the structure and the function of a text, and according to Riessman [22] "it interrogates both language and intention". One approach is to use a structural analysis that investigates how narratives are put together to achieve the narrators' aims. Moreover, Labov and Waletsky [23] maintain that a complete narrative consists of six elements (Table 1). In a structural analysis [24], stories with similar factual content can be compared to discern similarities and differences in how they are presented. Another type of narrative analysis is thematic in nature [22], examining stories as a whole to discover their functions.

**Table 1:** The narrative elements proposed by Labov and Waletsky [23], adjusted for the present study

Element	Sub-element	Description
Abstract		What the story is about

Orientation		Relevant background information

Complicating action		Sequential clauses providing chronology necessary for a narrative

Evaluation		Why the story was told, and the storytellers own opinions and values

	*External evaluation*	The storyteller expresses his or her opinion in explicit or implicit ways

	*Internal evaluation*	How the language is used to communicate values

	*Comparators*	Creating values by comparing what did and what did not happen

	*Extension device*	Connecting different episodes as if they were causally related

	*Explications*	The storytellers explications of what happened

	*Lexical signalling*	Use of strong, clearly evaluating words

Resolution		What happened in the end

Coda		Final remarks outside the story

**Figure 1:**
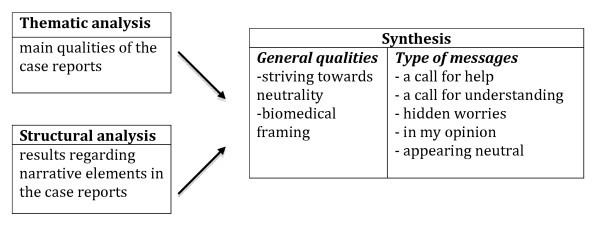
**Illustration of narrative analysis of the case reports**.

In accordance with our aim, we used a narrative approach based on thematic analysis [22], as well as structural analysis according to Labov and Waletsky [24]. The analyses of the case reports were performed along two parallel lines, one comprising a search for main qualities (thematic analysis) and the other a search for the contents of the narrative elements as proposed by Labov and Waletsky [24] (structural analysis), which agrees with the template style of qualitative analysis described by Crabtree and Miller [25]. In a subsequent step, the results of the two analyses were combined to create a final comprehensive description of the narrative features of the case reports (Figure 1).

#### Thematic analysis

Regarding main qualities, each story was considered in its entirety in order to find ways to relate to the problems experienced and the messages conveyed to the reader. This was done through repeated reading of the report and writing of short memos (i.e., a few words about prominent aspects).

Guided by the memos, and quite early in the readings, we identified five main qualities that clearly differed between the 19 case reports:

a. The certainty/uncertainty expressed by physicians regarding what help they needed to handle the cases in question; some physicians felt more or less insecure, while others stated how they wanted their case to be handled.

b. The physicians' descriptions of their emotional involvement in their cases.

c. The physicians' displaying of values and opinions.

d. The propensity to consider the cases from different angles.

e. Provision of contradicting facts.

#### Structural analysis

Slightly modified versions of the six narrative elements described by Labov and Waletsky (Table 1) were used in the structural analysis, which gave the following results:

*Abstract*: all the case reports were about problems related to sickness certification.

*Orientation*: the extent and type of background information about the individual patients and the medical interventions varied.

*Complicating actions*: only a few narratives provided a clear chronology.

*Evaluation*: a general feature was a striving to achieve neutrality in their writing about the patient, both in content and form, although the following narrative sub-elements of this aspect could be found:

*External evaluation*: the physicians sometimes expressed personal opinions or gave evaluating remarks.

*Internal evaluation*: there were only a few examples of words or expressions being repeated to further clarify something.

*Comparators*: none were found.

*Extension device*: in some cases, facts or episodes concerning the patient were presented together in a way that indicated contradictions.

*Explications*: the physicians' opinions or interpretations of what had happened were provided in some of the case reports.

*Lexical signalling*: there were some examples of the use of forceful adjectives, verbs, or nouns implying problematic situations.

*Resolution*: for obvious reasons, there were no complete stories showing how cases ended.

*Coda*: some of the case reports ended with explicit questions or remarks that outlined the problems the physicians had or what kind of advice they asked for.

### Synthesis

In the next step, the case reports were grouped into preliminary categories that we called "types of messages". This process emanated from both the main qualities of the narratives and the structural features identified according to Labov and Waletsky [23]. The way that the structural characteristics shed light on how the messages were formed is exemplified by how *evaluations *conveyed the emotional involvement and presence of the reporting physician. Through comparison within and between the preliminary categories, both the grouping and the names of the categories were adjusted until the categorization gave a fair representation of the data. This is illustrated by the definite categories "hidden worries" and "appearing neutral", which were designated together as "mere glimpses" in the early stages of this categorization. Although some case report had constituents from more than one category, it was easy to identify what type of message predominated in each case.

Finally, to determine whether the types of messages could be applied in a more general sense to this kind of data, we examined another 25 case reports, all but two of which came from the first sickness insurance course (the exceptions were the two reports included in the first analysis). In this work, the messages found were used as a framework for analysis.

Considering the roles of the authors, ME performed the initial analyses, and discussions were held with CER throughout that process. Towards the end of the work, the gross structure and the categories were also modified in response to suggestions from KA. All three authors agreed on all the details presented in the final results.

## Results

All the case reports on problematic sickness certification showed the following general qualities: the physicians strived towards neutrality in form and content, and they tended to interpret the patients' stories within a traditional biomedical frame. However, the narrative analyses revealed that, under the surface of this conformity, there were clear differences between the cases.

### Types of messages

Five different types of messages were identified, which described how the participating physicians wrote about problematic sickness certification cases. These messages were given the following names to illustrate what the physicians wanted to express and accomplish in the context of the sickness insurance courses: "a call for help", "a call for understanding", "hidden worries", "in my opinion", and "appearing neutral". Each of these is described in more detail below.

#### A call for help

"I'm telling you about something that is problematic, and I need your help"

These case reports were never in the form of mere medical charts; instead, they tended to be fairly long and more like complete stories, driven by what Labov and Waletsky [24] call *complicating actions*. The manner of presentation was vivid and personal, and the physicians tried to make themselves understood, expressing *external evaluations *such as "I felt divided about sickness certification from the beginning." One case report concerning a young woman with psychosocial problems entailed *lexical signalling *in that the word "heavy" was used five times on one page. The word "I" was used in many case reports, and the relationship to the patient was in some way always described essentially as illustrated by the following statement: "This is about a female PhD student that I met for the first time in December 2002." Different strategies to address the case and the physician's feelings of helplessness were often presented. The *coda *sometimes included an open request for help: "What should I do? Suggest half-time disability pension?"

#### A call for understanding

"I want you to understand that this is problematic"

This type of message was close to "a call for help", with obvious emotional involvement of the physician, but, in contrast to the previous category, there were more expressions of frustration than of helplessness. There were some *explications*, such as: "I believe that the employer wants her to be on sick leave for a long time." Multiple angles were seldom provided, and there were very few requests for advice about alternative ways to handle a case.

#### Hidden worries

"I'm describing something problematic while trying to remain indifferent"

Some of these case reports were structured as medical charts, with conventional headings like "background" and "status". The physician as a person was less visible, as was his/her relation to the patient. There was often a dramatic, or even absurd, touch that was created by presentation of contradicting episodes or facts. This represents *extension device*, which can be exemplified as follows: "on sick leave since 1997 ... lives in a house in the country, has nine dogs, does carpentry and other work." *Lexical signalling *also occurred, as shown by these words used to describe the case of a middle-aged man: "ongoing conflict.... diapers ...defecation-incontinence...unbearable ... gas himself to death ...ambulance ... not suicidal ... permanent disability pension ... thankful." Expressions of feelings, opinions, and values were not explicit, but there were some implicit *evaluations*: "A referral for psychotherapy ... was sent two years ago, still waiting for an appointment." Even information like "more backwards and forwards, and now ... 66% employment ... now a new certificate concerning health status" is clearly perceived as a type of evaluation by someone who is familiar with the subject; there were no obvious requests for help to solve the particular case used as an example here.

#### In my opinion

"I'm telling you my opinion about the problems"

In this type of message, the physician expressed involvement in the case, as well as thoughts about how she or he wanted the case to be handled, but there were no clear requests for help from others. *External evaluations *were sometimes present, as in the case of a 62-year-old woman who was diagnosed as depressed by the physician: "She is still fragile." and "She could recover if they find suitable work tasks for her." There was less mention of being frustrated, and multiple angles were not provided.

#### Appearing neutral

"I'm just describing"

These presentations were either short medical reports or actual copies of existing charts, and they comprised very few narrative elements except for aspects of *orientation*. The physician her-/himself was more of a spectator, since her/his relationship with the patient was not presented. Values and opinions were seldom visible, and there were no requests for help.

### Analysis for validation

In subsequent analysis of 25 additional case reports, the same types of messages were easily identified, and no new categories were recognized. Some case reports had constituents of more than one type, but it was never difficult to discern the main message.

## Discussion

The present narrative analysis focused on messages conveyed in the context of improvement courses, and it revealed five types of messages in 19 case reports that physicians had written to describe problems associated with sickness certification. The following messages were identified: "a call for help", "a call for understanding", "hidden worries", "in my opinion", and "appearing neutral". It became apparent that some of the messages conveyed by the physicians included openness and requests for help or understanding, whereas others were more restrained.

There is little knowledge on how physicians handle problems that arise in sickness certification cases, but our results can now give some guidance. Although the material used in this study was gathered in a specific context (i.e., as a pre-assignment for an improvement course), our findings have potentially relevant implications, because they concur with lines of thought and research findings presented in the literature.

### Facing the problem or not

In the majority of the case reports analysed in this study, the writers had obviously endeavoured to achieve neutrality in both content and form, an aspiration that also applies to case reports about patients written for purposes other than describing a specific problematic situation [26]. Many of the case reports included here showed similarities with texts in medical charts, and the physician as a person was more or less invisible. However, a striving for neutrality does not necessarily imply that the problem at hand is not acknowledged, although it does make that possibility less likely. If the physician, also in practice, responds to a problematic situation mainly by appearing neutral, there is a risk that ineffective and disease-oriented measures will be perpetuated [16]. There are links between neutrality beyond reason and medical education. In a textual analysis of essays written by medical students in the United Kingdom, Howe and colleagues [27] explored linguistic clues to what they called depth of apparent reflection, and they concluded that minimal use of first-person reflections might identify students who need further professional development. Medical education per se tends to condition what can be termed the biomedical reflex [28,29]. Such a reflex entails an immediate and disease-oriented framing of the presentation of symptoms that reduces the patient to his/her symptoms and physical signs by excluding the communicating student or the physician from the professional perspective. Accordingly, medical students may lose some of their empathic competence along the course of their education, since encouraging empathy requires the recognition of a subject who is open to the patient as a person [30]. Hence the limitation to empathy should be reflected in the ways that qualified physicians produce texts in their professional role. Aaslestad [31] has described how transforming the patient into an isolated medical text backwardly sets limits on the physician's relationship with the patient.

Charon [32,33] and other researchers [34,35] applying a narrative approach have presented findings indicating that using more reflective writing can strengthen medical students' empathic interaction with patients. When describing "the medical case" in writing, the process of making both the physician/student as a person and the relationship with the patient clearly visible may prove to be a challenging as well as an enriching exercise.

### The different messages

A general finding made in an investigation by Arborelius et al. [36] was that GPs do not usually allow themselves to show feelings of uncertainty in consultations that they have difficulty grasping. In contrast, according to Schön [19], reflective and successful practitioners permit themselves to experience surprise or confusion in situations that they find uncertain or unique. This makes the situation open to reframing, which allows the practitioner to find more productive ways of challenging the cause of the uncertainty. Extending practice into the writing of a report about a problematic case may enable a reframing of the situation in a manner that was not achievable while face to face with the patient. Malterud et al. [37] performed a study in which physicians were asked to write about a case in which their vulnerability in relation to a patient had been exposed, and the results obtained indicated that spontaneous revelation of emotions by the physician might lead to constructive interaction with the patient. Similarly, we found that the type of message that we refer to as "a call for help", which includes both emotions and a striving to realize different aspects involved, and to some degree even the physician's relationship with the patient, is likely to provide new perspectives on the case and also give the physician writing the report responses from colleagues. On the other hand, "a call for understanding" (i.e., asking for sympathy from colleagues) exposes a reluctant attitude towards reframing a problematic situation that one actually recognizes. In other words, it reveals ambivalence. If this were to recur more persistently in real practice, it could increase the burden of less effective practice.

By comparison, the emotional involvement in the type of message that we designate "in my opinion" is more defensive. It is assumed that the problem is outside of the doctor-patient interaction, which might be both correct and incorrect. If the physician tends to take on heavy responsibility for the patient in financial terms and/or regarding more general acceptance of the role of advocate [38], professional distance may be lacking. On the other hand, the message "hidden worries", which in some cases includes elements of irony or even cynicism, may reflect a non-professional distancing to the problems faced. It is worth noting that cynicism might be a sign of exhaustion or even burnout among physicians and other medical professionals [39,40]. This potential connection merits some attention, because it has been suggested that sickness certification is a work environment problem for physicians [7,8]. However, considering the physicians in our study, their problems often emerged during the role-play sessions and discussions conducted in the sickness insurance courses, also in cases corresponding to "appearing neutral" or "hidden worries". We learned that even these types of messages could be altered when challenged by involvement and curiosity.

In light of our findings regarding both neutrality as a general inclination and individual messages, it is possible that these aspects also have a bearing on physicians' ways of expressing and relating to clinical problems [1] other than those that concern sickness certification. This assumption is supported by the general character of the messages. It would be highly interesting to further investigate physicians concerning possible relationships between their reflection-in-action and the way they write about their problematic cases.

#### Methodological considerations

The participating physicians had different work environments, being employed in a primary care setting, an occupational health service, or a rehabilitation clinic. Nevertheless, considering the results of our earlier studies [16,17] of a larger sample of the same material, which used other analytical methods, and also our experiences during the sickness insurance courses, it does not seem that the different clinical backgrounds of the physicians affected the way that they presented problems in the case reports. However, the physicians knew that their texts would be read and discussed by smaller groups of colleagues. In this context, writing about a problematic case might have had different functions. For some, it might have provided a sense of belonging to a group of physicians who perceived sickness certification as being problematic [41]. In addition, some might have hoped for concrete aid in handling their cases. For example, it is possible that a physician will issue more "calls for help" when he/she knows that a particular patient will soon appear for a consultation. Other participants in our study may have been more reluctant to expose their problems and shortcomings. If that was indeed so, we do not regard it as a confounder in the interpretation of our findings, but rather as an example of one of the factors in medicine that may have an impact on how physicians present their cases.

The reports we analysed dealt exclusively with cases in which the participating physicians did not feel comfortable with their role in sickness certification. Reasonably, that created a larger span between the messages than if the sample had been made up of physicians' reports that were representative of sickness certification cases in general. In these case reports, "a call for help" was probably formulated more often and more expressively, while the attitudes of reluctance reflected as "in my opinion" and "appearing neutral" emerged in greater contrast. Still, we do not think that the selection of case reports in our study imposed attitudes on the participating physicians that were not, to any significant extent, originally theirs.

Researchers who study narratives advocate that the main strength of a narrative is its inherent subjectivity [26,32,42] and that the challenge is to capture aspects of this phenomenon as data in their context and interpret them appropriately. Our categorization of types of messages was based on this perspective. In this study, we performed a qualitative analysis of 19 case reports that were sampled from a much larger material. The two of us who carried out the actual analysis (ME and CER) had previously read the entire collection of 260 case reports, and both of us had also worked on most of them in role-plays and discussions in the sickness certification courses. Thus, we were very familiar with the material, and we believe that we possessed the necessary theoretical sensitivity [43] to analyse it. Notwithstanding, the second author (KA) provided external scrutiny that added clarity and communicability to the findings, and her agreement with the results strengthened them.

The fact that we created a predefined sample by no means implies that our aim was to achieve a statistically representative sample. On the contrary, we wanted to obtain a sample that contained a sufficiently varied selection of case reports. Since we found no new messages in the validation sample consisting of 25 case reports, we conclude that the material was adequately saturated [43,44] for the specific context of our study (i.e., sickness certification cases that physicians found to be problematic). We cannot judge whether our findings are transferable to problematic sickness certification consultations in real life. However, if writing about one's own practice is in any way correlated with the actual practice itself, we believe that our findings provide some ideas that can aid understanding of reflection-in-action in problematic sickness certification. Furthermore, the general character of the messages we detected suggests that they are also relevant for describing problematic situations other than sickness certification.

## Conclusions

The case reports analysed in this study showed that physicians write in clearly different ways about the problems they face when handling sickness certification cases. The descriptions ranged from being neutral and not mentioning the problems to displaying emotional involvement and asking for help. The general character of the messages suggests that they can also apply to case reports concerning problems in areas other than certification of sickness absence. If pertinent relationships can be discerned between reflection-in-practice and narrative writing about practice, it will provide an approach for further investigations of consultations that are perceived as problematic and also medical education. Moreover, concurrent use of thematic and structural analysis can extend the potential applications of narrative methodology in research on clinical practice.

## Competing interests

The authors declare that they have no competing interests.

## Authors' contributions

ME participated in the design of the study, collected the material, carried out the primary analyses, and drafted the manuscript. CER made substantial contributions to concepts and the design of the study, and participated in the analyses and interpretation of data. KA revised the design, results, and manuscript critically for important intellectual content. All authors read and approved the final manuscript.

## Authors' information

Our former experience of problematic sickness certification is from being GPs with a special interest in this area, both as teachers and researchers (CER and ME). We were group leaders at the courses and thereby had a pre-understanding of physicians' problems in handling such certification. The second author (KA) has clinical experience as hospital social worker and has educated physicians in insurance medicine and researched physicians' sickness certification practices over the last decades.

## Pre-publication history

The pre-publication history for this paper can be accessed here:

http://www.biomedcentral.com/1471-2296/12/18/prepub
